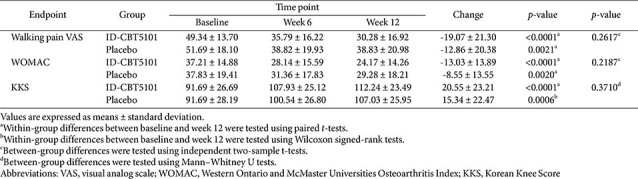# Randomized, Double-Blind, Placebo-Controlled Trial of ID-CBT5101, a Tyndallized *Clostridium butyricum* Postbiotic, in Adults with Mild-to-Moderate Knee Osteoarthritis

**DOI:** 10.4014/jmb.2512.12024

**Published:** 2026-01-22

**Authors:** Han Bin Lee, Jinho Lee, Kyuho Jeong, Jungwoo Yang, Young Hoon Jung, Jin Seok Moon

**Affiliations:** 1 Ildong Bioscience, Pyeongtaek-si 17957, Republic of Korea; 2 Ildong Pharmaceutical Co., Ltd., Seoul 06752, Republic of Korea; 3 Department of Microbiology, College of Medicine, Dongguk University, Gyeongju 38066, Republic of Korea; 4 School of Food Science and Biotechnology, Food and Bio-industry Institute, Kyungpook National University, Daegu 41566, Republic of Korea

**Keywords:** Osteoarthritis, Postbiotic, *Clostridium butyricum*, ID-CBT5101, Randomized controlled trial, Gut–joint axis

## Abstract

Osteoarthritis (OA) is a common cause of chronic pain and functional impairment in older adults. Evidence suggests a gut–joint axis, where gut dysbiosis and systemic low-grade inflammation may contribute to OA pathogenesis. Postbiotics, which are non-viable microbial products such as heat-killed bacteria, have been proposed as safe and stable alternatives to live probiotics. ID-CBT5101, a tyndallized *Clostridium butyricum* preparation, reduced inflammation and preserved cartilage in a rat OA model. We conducted a 12-week randomized, double-blind, placebo-controlled trial to assess ID-CBT5101 safety and efficacy in adults with mild-to-moderate knee OA. Ninety-six participants were randomized to receive either ID-CBT5101 (1.0 × 10^10^ CFU-equivalents/day) or placebo. The primary endpoint was the change from baseline in walking pain on a 100-mm visual analog scale (VAS). Secondary outcomes included WOMAC, Korean Knee Score (KKS), patient global assessment, and serum biomarkers. Both groups showed significant within-group improvements in VAS, WOMAC, and KKS over 12 weeks. However, no significant differences were observed between groups for any clinical endpoint. Serum interleukin-6 (IL-6), cartilage oligomeric matrix protein (COMP), prostaglandin E_2_ (PGE_2_), leukotriene B_4_ (LTB_4_), transforming growth factor-β (TGF-β), and high-sensitivity C-reactive protein (hs-CRP) showed no consistent changes favoring ID-CBT5101. Safety profiles were comparable between groups, with no treatment-related adverse events. ID-CBT5101 was safe and well-tolerated, but it did not demonstrate significant clinical efficacy compared with placebo.

## Introduction

Osteoarthritis (OA) is the most common form of arthritis and a major cause of pain and disability in older adults [1, 2]. Global Burden of Disease analyses estimate that OA affects hundreds of millions of individuals worldwide and imposes substantial socioeconomic and quality-of-life burdens [1, 2]. Knee OA is characterized by chronic pain, stiffness, and impaired function. Pathologically, OA involves cartilage degeneration, synovial inflammation, subchondral bone remodeling, and changes in periarticular structures. Modern concepts increasingly view OA as a whole-joint disorder rather than a simple “wear-and-tear” phenomenon [1].

Current pharmacological treatments for knee OA, such as nonsteroidal anti-inflammatory drugs and analgesics, primarily provide symptomatic relief and are limited by gastrointestinal, cardiovascular, and renal safety concerns, particularly with their long-term use. Disease-modifying OA drugs that appreciably alter joint structures or long-term disease trajectories remain elusive [1, 2]. In this context, there is growing interest in adjunctive strategies that target the upstream drivers of disease, including low-grade systemic inflammation and metabolic dysregulation.

Emerging evidence suggests that the gut microbiota and its metabolites can influence joint inflammation, cartilage catabolism, and pain perception through the gut–joint axis [3-7]. In animal models, perturbations in gut microbiota composition and increased intestinal permeability have been linked to heightened joint inflammation and pain behaviors, while specific microbial taxa and metabolites have been associated with protection against cartilage damage [3-5, 8-10]. Observational studies in humans have suggested that dysbiosis, metabolic endotoxemia, and diet-related changes in short-chain fatty acids (SCFAs) may correlate with OA severity and symptom burden [3, 5, 9, 10].

Both live probiotics and postbiotics (non-viable microbial preparations) have been explored as microbiome-targeted interventions for OA. Randomized trials of live lactic acid bacteria have reported improvements in knee pain, function, and inflammatory markers in selected OA populations [11]. In parallel, conceptual frameworks and consensus statements have defined postbiotics as “preparations of inanimate microorganisms and/or their components that confer a health benefit on the host” [12, 13]. Tyndallized, heat-killed bacteria preserve cell-surface structures while eliminating replication-competent organisms, which may improve safety and stability, particularly in older and immunocompromised individuals [12, 13].

*Clostridium butyricum* is an anaerobic, spore-forming, butyrate-producing bacterium that is used as a probiotic in several Asian countries. It supports intestinal barrier function and modulates immune responses in intestinal models [3-5, 8]. Preclinical data suggest that *C. butyricum* and its metabolites can influence the T helper 17/T regulatory cell balance, epithelial barrier integrity, and inflammatory signaling pathways relevant to both gut and joint homeostasis [3-5, 8, 14, 15].

ID-CBT5101 is a tyndallized preparation of *C. butyricum* IDCC 5101. In a previous study published in this journal, oral ID-CBT5101 attenuated pain behaviors, reduced synovial inflammation, and preserved cartilage structure in a monosodium iodoacetate-induced rat model of knee OA, accompanied by favorable changes in inflammatory mediators and cartilage-related markers [16]. In addition to ID-CBT5101, other *C. butyricum* preparations have also shown potential benefits in preclinical osteoarthritis models, including improvements in pain-related behaviors and/or structural and inflammatory readouts [17-20].

Based on these findings, we hypothesized that ID-CBT5101 may alleviate knee pain and modulate inflammatory pathways in human OA as a microbiome-targeted postbiotic intervention. Therefore, we designed a randomized, double-blind, placebo-controlled clinical trial as a translational extension of our preclinical study to evaluate the safety and exploratory efficacy of ID-CBT5101 in adults with mild-to-moderate knee OA. We aimed to determine whether the anti-inflammatory and chondroprotective effects observed in a rat model would translate into symptom relief and biomarker modulation in an unselected clinical OA population.

## Materials and Methods

### Study Design

This single-center, randomized, double-blind, placebo-controlled, parallel-group trial was conducted over 12 weeks in adults with symptomatic knee OA. This study adhered to the principles of the Declaration of Helsinki and the Good Clinical Practice guidelines. The protocol was approved by the Institutional Review Board of Kyung Hee University Medical Center (IRB No. 2015-06-105), and all participants provided written informed consent before any study procedures were performed.

The participants attended clinic visits at screening, baseline (week 0), week 6, and week 12. At each visit, prespecified clinical outcomes, safety parameters, and concomitant medications were assessed according to a standardized schedule. Randomization, data management, and statistical analyses were conducted independently of the sponsor.

### Participants

Eligible participants were men or women aged 40–75 years with radiographically confirmed knee OA (Kellgren–Lawrence grade I–III) in at least one knee and a history of knee pain for ≥ 3 months. At screening and baseline, they were required to have a minimum level of symptomatic pain, defined as a walking knee pain visual analog scale (VAS) score above a prespecified threshold, and to be able and willing to comply with the study procedures and visit schedules.

The key exclusion criteria were inflammatory arthritis (such as rheumatoid or psoriatic arthritis), crystal arthropathies, secondary OA due to major trauma or other causes, and prior arthroplasty of the index knee. Individuals with uncontrolled comorbidities (including severe cardiovascular, hepatic, renal, or psychiatric diseases), current malignancy, or significant gastrointestinal conditions that could interfere with oral absorption or gut microbiota (*e.g.*, inflammatory bowel disease) were excluded from the study. The use of systemic corticosteroids, intra-articular injections within a prespecified washout period, or other investigational products was not permitted.

### Randomization and Blinding

Participants who met all eligibility criteria at baseline were randomly assigned in a 1:1 ratio to receive ID-CBT5101 or a matching placebo for 12 weeks. An independent statistician generated the randomization sequence using a computer-based algorithm with permuted block sizes. The randomization list was concealed from the investigators, study staff, participants, and outcome assessors.

The study products were packaged in identical blister packs and labeled with unique randomization codes. The ID-CBT5101 and placebo tablets were indistinguishable in terms of appearance and taste. Allocation was performed by an unblinded pharmacist or a designated staff member who was not involved in the outcome assessment or data analysis. Blinding was maintained for the participants, investigators, and sponsor until the database was locked and the primary analysis was completed.

### Intervention

The investigational product, ID-CBT5101, is a tyndallized oral formulation of *C. butyricum* IDCC 5101, supplied as an 800-mg coated tablet. Each tablet contained 300 mg of tyndallized *C. butyricum*, corresponding to 5.0 × 10^9^ colony-forming units (CFU) before inactivation, along with pharmacologically inactive excipients (lactose, corn starch, microcrystalline cellulose, fructooligosaccharide, carboxymethyl cellulose calcium, dicalcium phosphate, magnesium stearate, hydroxypropyl methylcellulose, ethyl vanillin, and a film coating). The manufacturing process ensured the absence of viable cells after the heat treatment.

Participants randomized to ID-CBT5101 were instructed to take one tablet twice daily, approximately 30 min before breakfast and 30 min before dinner, for a total daily dose of 1.0 × 10^10^ CFU-equivalents. The placebo group received tablets that were identical in appearance and composition but did not contain *C. butyricum*. All study products were manufactured and packaged according to the relevant quality and regulatory standards.

Adherence was assessed using tablet counts and participant diaries collected at each visit. The participants were encouraged to maintain their usual diet and physical activity during the study period. Concomitant OA therapies, including analgesics and nonsteroidal anti-inflammatory drugs, were managed according to predefined rules and carefully documented at each visit.

### Outcome Measures

Efficacy and safety assessments were conducted at baseline (week 0), 6 weeks, and 12 weeks. The primary efficacy endpoint was the change in walking knee pain in the target knee from baseline to week 12, measured using a 100-mm VAS. Participants rated their average walking pain over the preceding week by placing a mark on the scale, with 0 mm representing “no pain” and 100 mm representing “worst imaginable pain.”

Secondary clinical endpoints were selected to capture the broader aspects of OA symptoms and function. The WOMAC was used to assess pain, stiffness, and physical function, and the primary WOMAC variable was the change in the total score from baseline to week 12. The overall knee status was evaluated using the Korean Knee Score (KKS), and the change in the total KKS from baseline to week 12 was analyzed. The patients’ global perception of treatment response was assessed using the patient global assessment (PGART), and the percentage change in PGART grades between weeks 6 and 12 was examined. Additional functional and quality-of-life outcomes were analyzed as prespecified in the protocol.

Exploratory analyses included a subgroup evaluation of participants with baseline walking pain VAS ≥ 30 mm, representing individuals with at least moderate knee pain at entry.

### Biomarker Assessments

To explore the potential systemic effects of ID-CBT5101 on inflammation and cartilage or bone metabolism, fasting blood samples were collected at baseline and week 12. Serum concentrations of interleukin-6 (IL-6), cartilage oligomeric matrix protein (COMP), prostaglandin E_2_ (PGE_2_), leukotriene B_4_ (LTB_4_), transforming growth factor-β (TGF-β), and high-sensitivity C-reactive protein (hs-CRP) were measured as exploratory biomarkers. All samples were obtained in the morning and analyzed at a central clinical laboratory using validated immunoassays, following the manufacturer’s instructions. Samples from the same participant were analyzed in the same batch to minimize inter-assay variability.

### Safety Assessments

Safety was evaluated throughout the trial by recording adverse events (AEs), performing physical examinations, measuring vital signs (blood pressure, pulse rate, and body temperature), and conducting laboratory tests (hematology, serum chemistry, and urinalysis). AEs were documented in terms of onset, duration, severity, outcome, and the investigator’s assessment of their relationship with the study treatment. Serious AEs and AEs that led to treatment discontinuation were identified and reviewed. Pregnancy testing was performed at screening for women of childbearing potential and repeated during the trial if clinically indicated.

### Statistical Analysis

Three analysis sets were defined before unblinding: the safety set, full analysis set (FAS), and per-protocol set (PPS). The safety set included all participants who ingested at least one dose of the study product and was used for safety analyses. The FAS, which served as the primary population for efficacy analyses, included all randomized participants who received at least one dose of the study product and underwent both a baseline and at least one post-baseline efficacy assessment. For FAS participants who lacked data at a given follow-up time point (*e.g.*, week 12), the last observation carried forward (LOCF) method was used to impute missing values. The PPS was defined as the subset of FAS participants who completed the 12-week intervention without major protocol violations, use of prohibited medications, or marked non-compliance (<80% or >120%), and was used for supportive efficacy analyses.

Continuous variables are summarized as the number of observations, mean, standard deviation (SD), median, and minimum and maximum values. Categorical variables are expressed as counts and percentages. Between-group comparisons at baseline and for changes from baseline were performed using independent two-sample *t*-tests for approximately normally distributed variables or Mann–Whitney *U* tests when normality assumptions were not met. Within-group changes from baseline were examined using paired *t*-tests or Wilcoxon signed-rank tests, as appropriate. When clinically relevant baseline imbalances were observed for a given endpoint, an analysis of covariance with the baseline value as a covariate was conducted as a supplementary analysis.

All statistical tests were two-sided, and a *p*-value of <0.05 was considered statistically significant. Statistical analyses were performed using SAS software (version 9.4; SAS Institute Inc., USA).

## Results

### Participant Disposition and Baseline Characteristics

Of the 103 individuals screened, 96 met the eligibility criteria and were randomized to the ID-CBT5101 (*n* = 48) or placebo (*n* = 48) groups. One participant in the ID-CBT5101 arm did not receive any of the study products, yielding a safety set of 95 participants (ID-CBT5101: *n* = 47; placebo: *n* = 48).

The FAS comprised 89 participants with at least one post-baseline efficacy assessment (ID-CBT5101, *n* = 44; placebo, *n* = 45). The PPS included 66 participants who completed the 12-week intervention without major protocol deviations (ID-CBT5101: *n* = 33; placebo: *n* = 33). Participants were excluded from the PPS primarily due to protocol violations, low compliance, prohibited use of concomitant medications, or withdrawal of consent. The mean treatment compliance exceeded 90% in both groups.

**Fig. 1:**
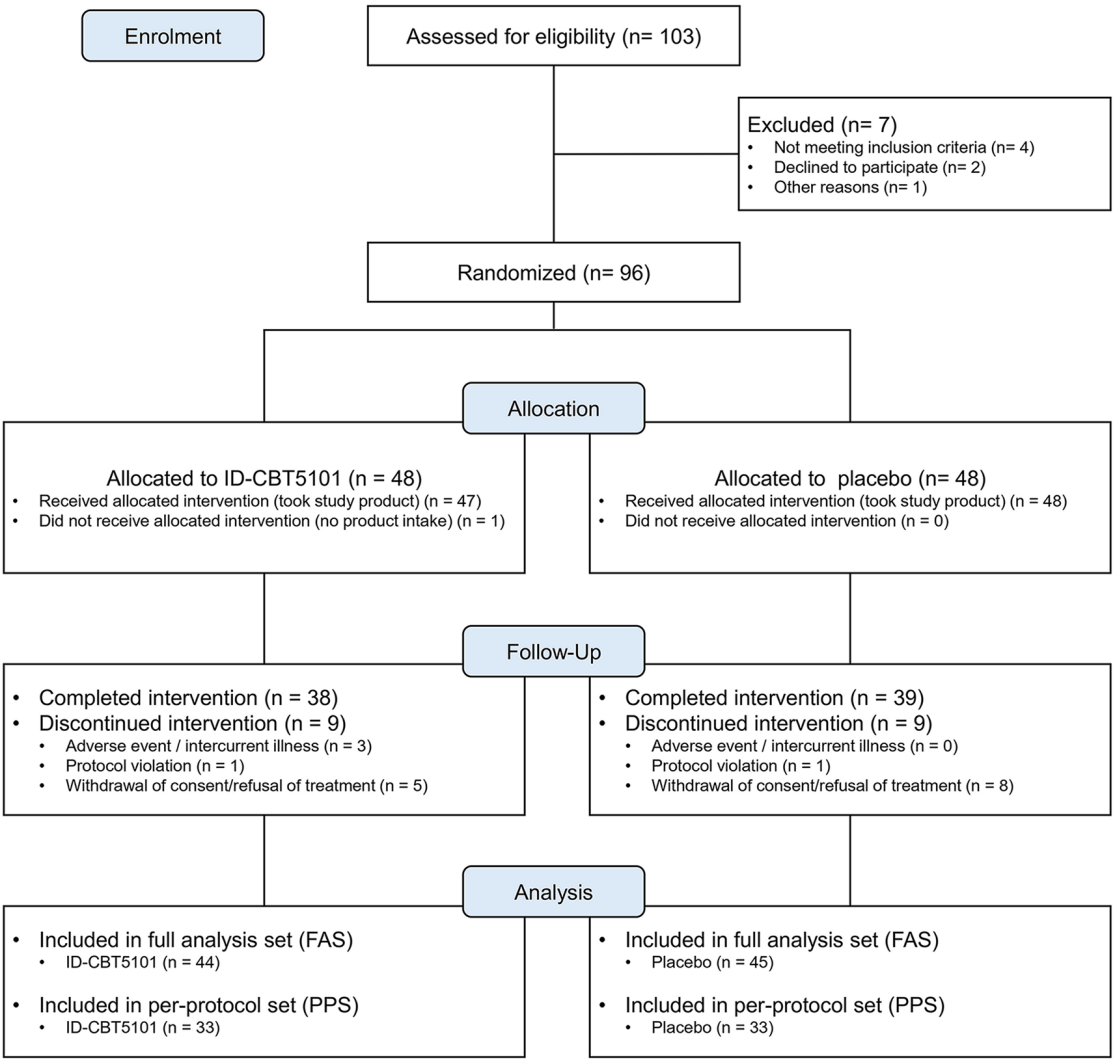
CONSORT flow diagram for the randomized, double-blind, placebo-controlled trial of ID-CBT5101 in adults with mild-to-moderate knee OA.

The baseline demographic and clinical characteristics were well balanced between the groups (Table 1). Participants were mostly in their early 60s, and there were no meaningful between-group differences in sex distribution, anthropometric variables, Kellgren–Lawrence grade, lifestyle factors, or baseline walking pain VAS, WOMAC, or KKS. Detailed baseline characteristics and participant flow are summarized in Table 1 and Fig. 1.

### Primary Outcome: Walking Pain VAS

In the FAS, the mean baseline walking pain VAS scores were 39.82 ± 17.83 mm in the ID-CBT5101 group and 39.13 ± 22.95 mm in the placebo group. Both groups showed a progressive reduction in walking pain over 12 weeks. At week 12, the mean VAS scores were 27.27 ± 17.88 mm with ID-CBT5101 and 30.42 ± 22.07 mm with placebo.

Within-group changes from baseline to week 12 were statistically significant in both arms (ID-CBT5101: −12.55 ± 21.71 mm, *p* = 0.0004; placebo: −8.71 ± 18.85 mm, *p* = 0.0034). However, the difference in the mean change between the groups was small and insignificant (*p* = 0.3757). In other words, in this cohort with relatively mild baseline pain, ID-CBT5101 did not demonstrate clear superiority over placebo in terms of the primary outcome (Table 2).

**Fig. 2:**
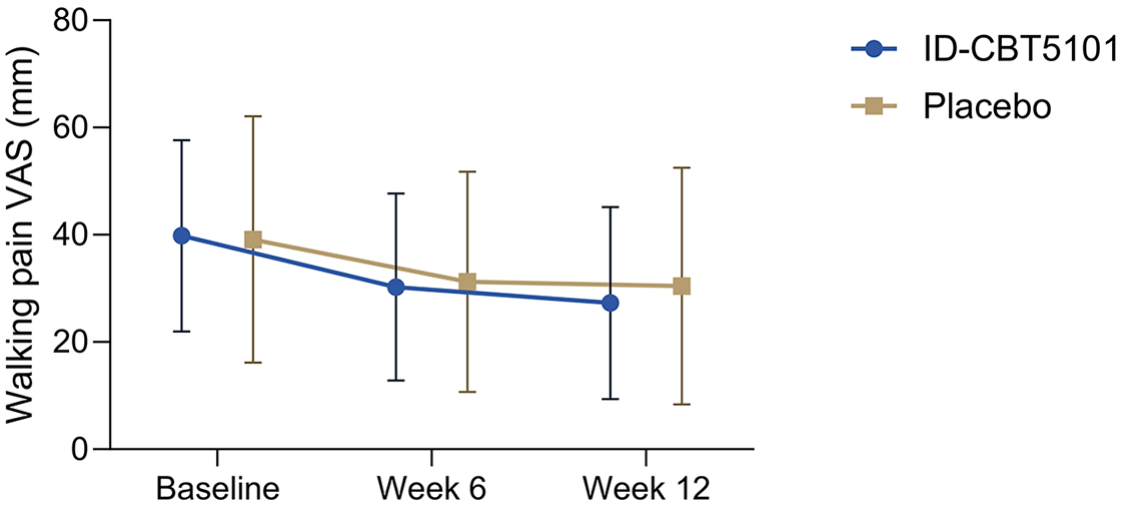
Changes in walking pain VAS scores over 12 weeks in the FAS. Mean walking pain VAS scores (0–100 mm) at baseline, week 6, and week 12 are shown for participants receiving ID-CBT5101 (1.0 × 10^10^ CFU-equivalents/day) or placebo. Data are presented as mean ± SD in the FAS, with missing values imputed using the last observation carried forward method.

The PPS results were similar. Baseline VAS scores were 38.52 ± 17.32 mm in the ID-CBT5101 group and 42.94 ± 23.43 mm in the placebo group, decreasing to 25.55 ± 17.22 mm and 30.61 ± 22.56 mm, respectively, at week 12. Mean changes were −12.97 ± 22.52 mm and −12.33 ± 17.31 mm (*p* = 0.8980). The VAS trajectories over time were largely parallel between the groups (Fig. 2).

### Secondary Clinical Outcomes

**WOMAC total score.** The WOMAC total scores improved in both groups. In the FAS, the baseline scores were 29.66 ± 16.68 (ID-CBT5101) and 30.38 ± 19.36 (placebo) and decreased to 21.07 ± 14.21 and 23.36 ± 17.69, respectively, at week 12. Within-group reductions were significant (ID-CBT5101: −8.59 ± 14.21, *p* = 0.0002; placebo: −7.02 ± 12.55, *p* = 0.0005), but the between-group difference in the mean change was not significant (*p* = 0.5822). The PPS results were consistent with these findings (Table 3).

**Korean knee score.** The KKS total scores increased over time in both treatment arms, indicating an improved overall knee status. In the FAS, the baseline KKSs were 103.68 ± 28.56 (ID-CBT5101) and 102.36 ± 28.05 (placebo), which increased to 118.05 ± 23.38 and 115.98 ± 26.91, respectively, at week 12. Within-group improvements were significant (ID-CBT5101: +14.36 ± 22.64, *p* = 0.0001; placebo: +13.62 ± 21.82, *p* < 0.0001), and the difference in the between-group change was not significant (*p* = 0.9118).

### Patient Global Assessment and Other Outcomes

The PGART scores remained relatively stable with modest changes in both groups. In the FAS, the mean PGART scores at week 6 were 2.81 ± 0.86 (ID-CBT5101) and 2.86 ± 0.77 (placebo), and at week 12 were 2.78 ± 0.92 and 2.90 ± 0.80, respectively. The between-group differences at weeks 6 (*p* = 0.8802) and 12 (*p* = 0.4592), as well as the percentage changes between weeks 6 and 12, were not significant (Table 3). Other patient-reported outcomes and functional measures showed within-group improvement, but no consistent pattern favoring ID-CBT5101 over placebo.

### Serum Biomarkers

Baseline levels of interleukin-6 (IL-6), cartilage oligomeric matrix protein (COMP), prostaglandin E_2_ (PGE_2_), leukotriene B_4_ (LTB_4_), transforming growth factor-β (TGF-β), and high-sensitivity C-reactive protein (hs-CRP) were similar between the groups in both the FAS and PPS. Over 12 weeks, changes in IL-6, COMP, PGE_2_, LTB_4_, and TGF-β were small and did not favor ID-CBT5101 in the between-group comparisons.

Baseline hs-CRP levels were low in both arms. Both groups exhibited decreases over 12 weeks, with a numerically greater reduction in the placebo group (−0.07 ± 2.15 vs. −0.40 ± 1.66 mg/L; *p* = 0.0168). Given the low absolute levels, lack of consistent changes in other markers, and absence of a corresponding clinical disadvantage, this isolated difference was considered unlikely to represent a meaningful safety concern. The biomarker data are summarized in Table 4.

### Subgroup Analysis: Baseline Walking Pain VAS ≥ 30 mm

A prespecified subgroup analysis was conducted in participants with baseline walking pain VAS ≥ 30 mm, representing individuals with at least moderate knee pain at study entry. In this subgroup, walking pain VAS, WOMAC, KKS, PGART, and serum biomarkers were re-analyzed to explore whether ID-CBT5101 might show greater clinical relevance in patients with a higher baseline symptom burden.

**Fig. 3:**
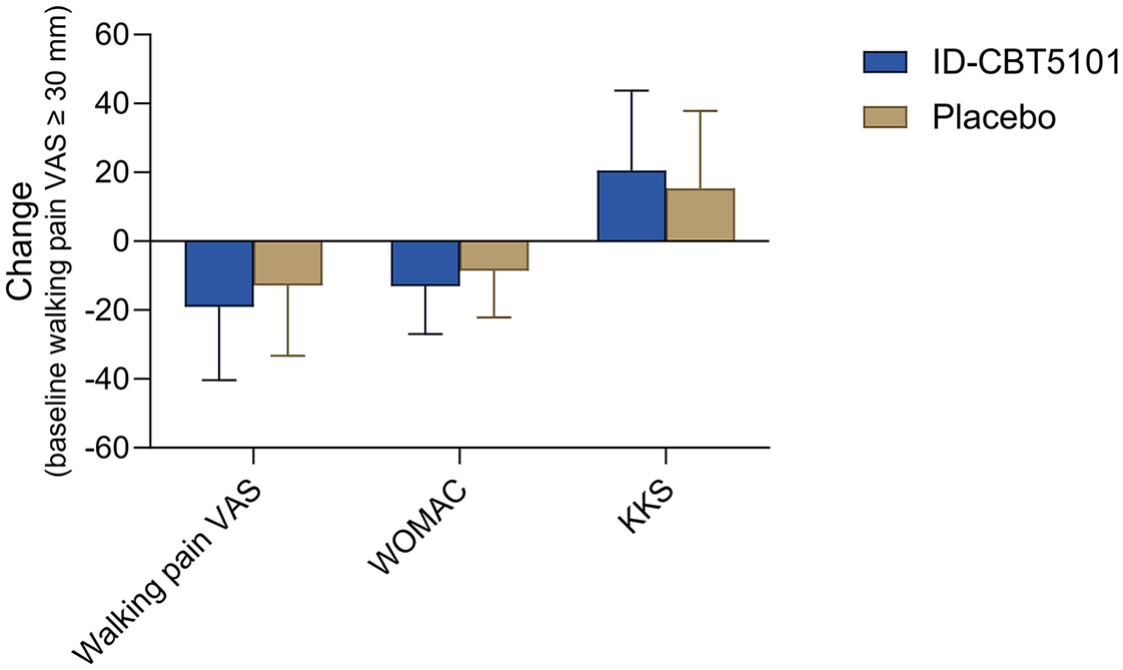
Subgroup analysis of clinical outcomes at week 12 in participants with baseline walking pain VAS ≥ 30 mm. Mean changes from baseline to week 12 in walking pain VAS (mm), WOMAC total score, and KKS total score are shown for the FAS subgroup with baseline walking pain VAS ≥ 30 mm. Negative values for VAS and WOMAC indicate improvement in pain and function, whereas positive values for KKS indicate functional improvement. Data are expressed as mean ± SD, with missing values imputed using the last observation carried forward method.

In the FAS subgroup, both treatment arms showed significant within-group reductions in walking pain VAS scores over 12 weeks, and ID-CBT5101 produced numerically greater improvements than the placebo. For example, the mean changes in walking pain VAS were −19.07 ± 21.30 mm with ID-CBT5101 and −12.86 ± 20.38 mm with the placebo. However, none of the between-group differences in change achieved statistical significance, likely reflecting limited power and variability in the response. Similar patterns were observed for the WOMAC, KKS, and PGART (Table 5 and Fig. 3).

### Safety and Tolerability

Overall, ID-CBT5101 was well tolerated. The incidence of treatment-emergent AEs was similar between the ID-CBT5101 and placebo groups in the safety set. Most AEs were mild or moderate and resolved without any sequelae. Gastrointestinal symptoms, upper respiratory infections, and musculoskeletal complaints were among the most frequently reported events, with comparable distributions between the groups.

A few serious AEs, including cerebral infarction and scrub typhus, occurred during this trial. The investigators judged these events to be unrelated to the study treatment. One participant in the ID-CBT5101 group discontinued treatment because of an AE. No deaths or serious treatment-related AEs were reported.

Vital signs and routine laboratory parameters exhibited no clinically meaningful trends or between-group differences, although some parameters exhibited small statistical fluctuations over time that were not considered clinically significant. Taken together, these findings support a favorable placebo-like safety profile for ID-CBT5101 in this OA population.

## Discussion

In this randomized, double-blind, placebo-controlled clinical trial involving adults with mild-to-moderate knee OA, 12 weeks of treatment with the tyndallized *C. butyricum* postbiotic ID-CBT5101 did not demonstrate statistically significant superiority over the placebo in improving walking pain VAS score, which was the primary endpoint. While both treatment arms showed significant within-group improvements in VAS, WOMAC, and KKS, the magnitude of change was comparable between the groups across both the FAS and PPS populations. Similarly, no consistent advantages were observed for systemic inflammatory or cartilage-related biomarkers following ID-CBT5101 treatment.

These results differ from previous preclinical findings in the monosodium iodoacetate-induced rat OA model, where ID-CBT5101 reduced pain behaviors and inflammatory markers and preserved cartilage integrity [16-18]. This translational gap highlights the common challenges in bridging animal model efficacy and human outcomes, particularly in complex diseases such as OA. Contributing factors may include differences in disease heterogeneity, comorbidities, background medications, and variability in the human gut microbiome.

The substantial placebo effects observed in this trial are consistent with the patterns observed in other OA studies and likely reflect multiple factors, including symptom fluctuation, non-specific benefits from trial participation, and background management optimization. A predefined subgroup analysis in participants with baseline walking pain VAS ≥ 30 mm suggested a trend toward greater pain reduction in the ID-CBT5101 group, but this was not statistically significant and should be interpreted cautiously.

Several mechanistic considerations may explain the lack of detectable treatment effects observed in this study. Notably, we did not assess fecal microbiota composition, intestinal permeability, or SCFA profiles, making it unclear whether ID-CBT5101 induces meaningful shifts in the gut environment [21, 22]. Furthermore, the dose and treatment duration, although informed by preclinical studies [18], may have been insufficient to affect downstream inflammation in this relatively unselected patient cohort. The biomarker panel used, which focused on systemic inflammation, may also have missed localized joint effects.

Postbiotics, such as ID-CBT5101, offer theoretical advantages, especially in older populations, owing to their non-viable nature and stability. By eliminating the risk of live bacterial colonization while preserving the immunologically active components [12, 13, 23], tyndallized formulations represent a potentially safe adjunctive approach. In this study, ID-CBT5101 demonstrated a favorable safety and tolerability profile with no treatment-related safety concerns identified.

Although this study did not demonstrate clinical efficacy, it provided useful data for refining future approaches to microbiome-targeted interventions for musculoskeletal diseases. Overall, in this single-center trial, 12 weeks of treatment with ID-CBT5101 was safe and well tolerated in adults with mild-to-moderate knee OA but was not superior to placebo in improving walking pain VAS, WOMAC, and KKS, or systemic inflammatory and cartilage-related biomarkers in an unselected patient population. These findings illustrate both the promise and the complexity of translating microbiome-targeted postbiotics from controlled preclinical models to real-world OA care settings.

Our findings support the need for integrated study designs that incorporate comprehensive microbiome and metabolome assessments alongside clinical endpoints and joint-specific evaluations. Stratifying participants based on markers of systemic inflammation, metabolic status, or dysbiosis patterns may help reduce heterogeneity and improve signal detection. Additionally, combining postbiotics with adjunctive strategies such as prebiotics, probiotics, or diet-based modulation warrants further investigation. Future trials should also evaluate long-term outcomes, including structural endpoints, to clarify the role of *C. butyricum*-based postbiotics in OA and related musculoskeletal conditions. Concurrently, the favorable safety profile observed suggests that such formulations may still be considered as components of broader gut–joint axis–oriented strategies for joint health, particularly in populations where tolerability and safety are paramount.

This study has some limitations. The single-center design and modest sample size may restrict generalizability and reduce the statistical power of the subgroup and biomarker analyses. The 12-week treatment period may not have been sufficiently long to observe meaningful structural or functional changes. Moreover, we relied on systemic biomarkers rather than joint-localized measures, such as synovial fluid analysis or imaging. Finally, we did not quantify rescue medication use, which may have affected the symptom outcomes.

Despite these limitations, this study has several strengths. A rigorous randomized controlled trial design, prespecified analysis sets, and detailed safety monitoring contributed to its robustness. To our knowledge, this is one of the first clinical evaluations of a *C. butyricum*-based postbiotic in OA and adds important neutral findings to the emerging body of evidence, helping mitigate publication bias in the field.

## Figures and Tables

**Table 1:** Baseline demographic and clinical characteristics of study participants in the full analysis set.

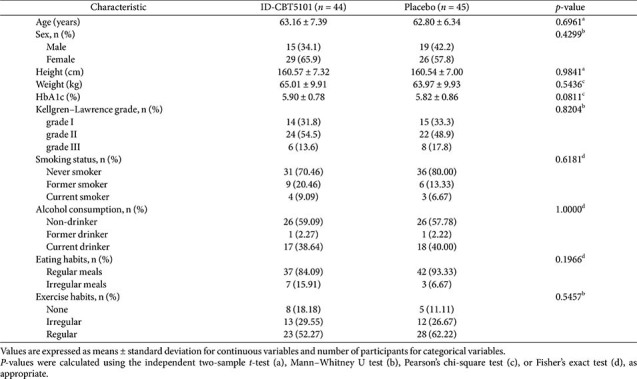

**Table 2:** Changes in walking pain visual analog scale (VAS) scores over 12 weeks in the full analysis set and per-protocol set.

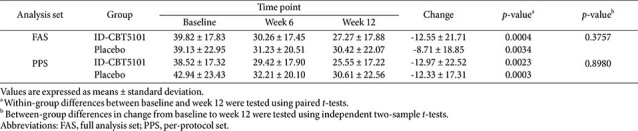

**Table 3:** Changes in WOMAC, Korean Knee Score, and patient global assessment over 12 weeks in the full analysis set and per-protocol set.

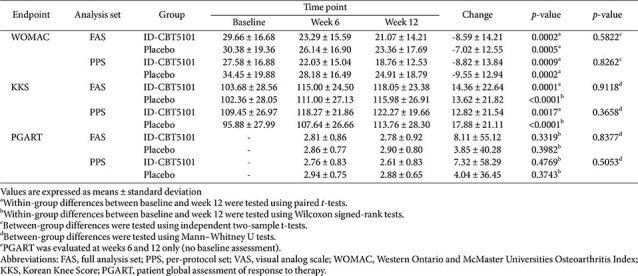

**Table 4:** Changes in serum inflammatory and cartilage-related biomarkers over 12 weeks (full analysis set).

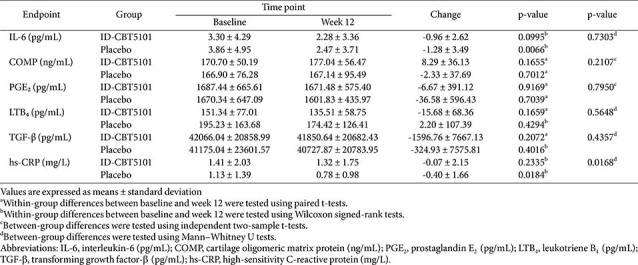

**Table 5:** Changes in walking pain VAS, WOMAC, Korean Knee Score, and patient global assessment over 12 weeks in baseline walking pain VAS ≥ 30 mm subgroup of the full analysis set.